# The Impact of Norovirus on Children and Adolescents: Implications for Ongoing Vaccine Development

**DOI:** 10.1007/s40124-025-00355-9

**Published:** 2025-08-13

**Authors:** Ming Tan

**Affiliations:** 1 Division of Infectious Diseases, Cincinnati Children’s Hospital Medical Center, Cincinnati, OH 45229 USA; 2 Department of Pediatrics, University of Cincinnati College of Medicine, Cincinnati, OH 45229 USA

**Keywords:** Norovirus, Diarrhea, Acute gastroenteritis, Vaccine, Children, Adolescent

## Abstract

**Purpose of Review:**

This article highlights the heightened vulnerability of children and adolescents to norovirus infection and discusses the implications for ongoing vaccine development efforts.

**Recent Findings:**

Young children remain highly vulnerable to norovirus infections and are at an increased risk of severe disease. However, a recent Phase 2b clinical trial in infants failed to demonstrate meaningful protection or other clinical benefits. Similarly, low protective efficacy was observed in adults in a separate Phase 2b clinical evaluation, raising concerns about the complexity of norovirus vaccine development, particularly for young children.

**Summary:**

Norovirus continues to pose a significant global health threat, affecting individuals of all ages and contributing to considerable morbidity, mortality, and socioeconomic burden. Children and adolescents are especially vulnerable, as they often spend time in closed or semi-closed environments such as daycare centers and schools, settings commonly associated with norovirus outbreaks. The lack of an FDA-approved norovirus vaccine underscores the urgent need for effective preventive strategies. Several vaccine candidates, utilizing diverse platforms, have advanced to Phase 2 clinical trials and beyond, targeting various age groups. While some have shown promise in adults, a recent Phase 2b clinical trial of a VLP-based vaccine in infants failed to demonstrate sufficient efficacy, and another Phase 2b study of an adenovirus-vectored vaccine reported low efficacy in adults. These findings suggest that novel approaches and strategies may be required to overcome the current challenges in norovirus vaccine development.

## Introduction

Noroviruses are non-enveloped RNA viruses with a single-stranded, positive-sense RNA genome. They belong to the *Norovirus* genus within the *Caliciviridae* family. Norovirus is the primary etiological agent of both epidemic and sporadic acute gastroenteritis worldwide, characterized by vomiting and explosive diarrhea, and affects individuals across all age groups. It is highly contagious, with an estimated basic reproductive number (R_0_) greater than 2 [1], is primarily transmitted via the fecal–oral route. The virus can spread rapidly through contaminated foods, water, surface, and even aerosols, often leading to large outbreaks, particularly in closed and semi-closed environments, such as cruise ships, military camps, restaurants, hospitals, long-term care facilities, and settings frequented by children and adolescents, including childcare centers, daycare facilities, and schools.

In addition to outbreaks, norovirus is a common cause of sporadic diarrhea in community settings. While most infections are self-limited and mild, severe disease can occur, especially in vulnerable populations such as infants, older adults, and immunocompromised individuals. Currently, treatment options for severe norovirus-associated illness are limited to rehydration and supportive care.

Our understanding of norovirus biology remains limited, largely due to the inability to propagate the virus in conventional cell culture systems. Although the human intestinal enteroid model, which mimics the human intestinal environment, can support limited replication of certain norovirus strains [2, 3], it is not yet widely scalable. Similarly, the pathogenesis of norovirus-associated disease is not fully understood, primarily owing to the lack of a suitable small animal model that accurately reflects human infection. Neonatal gnotobiotic pigs can be infected with certain genogroup II (GII) strains under germ-free conditions and may develop limited diarrhea [4], but this model has notable limitations.

The absence of a reliable culture and robust small animal model has also hindered progress in understanding norovirus immunology and in developing effective vaccines. Additionally, host genetic factors, such as the genes expressing the critical enzymes in the biosynthesis pathway of histo-blood group antigens (HBGAs) [5], which act as susceptibility factors for norovirus infection, along with the high prevalence of asymptomatic infections, further complicate the study of disease outcomes.

This review summarizes recent advances in norovirus research, with a focus on children and adolescents as vulnerable populations and explore the implications of these findings for current vaccine development efforts.

## Burden of Norovirus Disease in the General Population

Norovirus infection poses a significant global public health challenge [6], causing an estimated 685 million cases of acute gastroenteritis annually, accounting for approximately 18% of all diarrheal disease cases worldwide [7, 8]. In low- and middle-income countries, the estimated prevalence of norovirus among patients with acute gastroenteritis is around 17% [9]. A systematic analysis [7] identifies norovirus as the leading cause of diarrheal illness across all age groups globally, responsible for an estimated 212,000 deaths each year, with approximately 99% of these occurring in low- to middle income countries.

In high-income countries, norovirus is also recognized as a major pathogen of diarrheal illness, including foodborne outbreaks and community–acquired infections. However, mortality rates are substantially lower in these settings. In the United States, the Centers for Disease Control and Prevention (CDC) estimates that norovirus causes 19 to 21 million illnesses, 2.3 million outpatient clinic visits, 465,000 emergency department visits, 109,000 hospitalizations, and approximately 900 deaths per year, in addition, norovirus is responsible for 58% of foodborne diarrhea cases acquired in the U.S. [10]. In contrast, corresponding data from developing countries remain less well-defined.

Norovirus is the most common cause of acute gastroenteritis outbreaks in the general population. These outbreaks frequently occur in healthcare facilities, educational institutions, cruise ships, military installations, and food service establishments, where the virus can easily spread through contaminated food, surfaces, or person-to-person contact. According to the Norovirus Sentinel Testing and Tracking (NoroSTAT) network, developed by the CDC in collaboration with 14 state health departments, 2,630 norovirus outbreaks were reported in the U.S. between August 1, 2024, and June 4, 2025 [11]. Notably, this number substantially exceeds the range observed during the same period in the 2012–2020 and 2021–2024 seasons [11, 12].

The surge in norovirus outbreaks during the 2024/2025 season may be attributed to the emergence of a new strain, GII.17[P16] [13], which accounted for approximately 75% of outbreaks that season [14, 15]. This new GII.17 strain that was formerly uncommon may have caught populations unprepared and rapidly rose to dominance. Similar increases in norovirus activity associated with the GII.17 strain were also observed around the world during the same period [12, 16, 17].

Norovirus outbreaks exhibit a clear seasonal pattern, with peak activity typically occurring during the winter months, from October through March. Due to this seasonality, norovirus is often referred to as the “winter vomiting bug”, and norovirus-related illnesses are commonly called “stomach flu”.

Beyond its health impact, norovirus also imposes a substantial economic burden. Globally, it is estimated to cause approximately $4.2 billion in direct healthcare costs and $60.3 billion in broader societal costs each year [18]. in the United States alone, foodborne norovirus infections cost around $2 billion annually, primarily due to healthcare expenses and lost productivity.

## Children’s Vulnerability to Norovirus Infection

Diarrheal diseases remain the second leading cause of death among children under five globally, accounting for approximately 1.7 billion cases and 525,000 deaths each year [19]. While norovirus can infect individuals of all ages, children, particularly those under 5 years old, experience the highest incidence rates [20]. Severe disease outcomes are most common in this age group, with 20.4 medically attended episodes per 1,000 person-years. This rate is significantly higher than the 4.5 episodes per 1,000 person-years observed among older adults aged 65 and above, who represent the second most vulnerable population [21]. This trend aligns with a recent study on norovirus disease in the first 2 years of life, which show that 33% of acute gastroenteritis cases in this age group were moderate or severe [22]. Birth cohort studies indicated that up to 90% of children in developing countries experience at least one norovirus infection, and as many as 70% develop norovirus-associated acute gastroenteritis during early childhood [23].

According to the World Health Organization (WHO), norovirus is responsible for 200 million episodes of diarrhea annually among children under five, resulting in 50,000 deaths, primarily in economically disadvantaged countries [24]. In the United States alone, norovirus is estimated to cause around 4.2 million diseases; 815,000 outpatient visits; 130,000 emergency department visits; 24,600 hospitalizations; and 38 deaths among children each year [20]. These data highlight the significant global public health burden of norovirus-related illness in the pediatric population, particularly in developing countries, where it contributes substantially to both morbidity and mortality among young children.

## Norovirus Prevalence in Adolescents

Educational institutions, including schools, summer camps, colleges, and universities, are frequently affected by norovirus outbreaks due to the virus's highly contagious nature, the close proximity of students and staff, and the widespread use of shared spaces and surfaces [25, 26]. These environments facilitate the rapidly spread of norovirus, often leading to outbreaks [27]. As a result, adolescents are considered a vulnerable population, although their incidence rates and disease outcomes are generally lower than those observed in young children attending childcare centers. Notably, norovirus was first identified during an investigation of an acute gastroenteritis outbreak at an elementary school in Norwalk, Ohio, USA, in 1968 [28, 29]. As the prototype strain, the virus was named GI.1 Norwalk virus, after this location.

In the United States, 2,383 norovirus outbreaks were reported in schools and childcare facilities between 2009 and 2019, resulting in 110,190 illnesses, according to the National Outbreak Reporting System (NORS) [30]. Norovirus was identified as the most common cause of acute gastroenteritis in schools. A review by Matthews and colleagues, summarizing norovirus outbreaks reported in the literature from 1993 to 2011, found that 10% occurred in educational institutions, including colleges, universities, and schools [31]. In Shanghai, China, 215 norovirus outbreaks were reported during 2016/2017 season, with 189 (87.9%) occurring in schools and kindergartens [32].

Another review of norovirus outbreaks in colleges and universities between 1985 and 2017 estimated that 61% were spread through person-to-person transmission, while 57% were linked to contaminated food. Higher attack rates were associated with smaller on-campus population sizes [25]. Norovirus outbreaks often result in temporary school closures for deep cleaning and disinfection. General guidelines to prevent or mitigate the impact of such outbreaks on campus emphasize the importance of good hygiene practices, excluding symptomatic individuals, particularly food workers in school cafeterias and dining halls, and thoroughly cleaning and disinfecting shared surfaces.

## Current Clinical Advancements in Norovirus Vaccine Development

Since the introduction of commercial rotavirus vaccines in 2006, now universally recommended by the WHO, the incidence of rotavirus-associated diarrhea disease has significantly declined. As a result, norovirus has emerged as the leading cause of pediatric gastroenteritis requiring medical attention in many countries. This shift further highlights the urgent need for a norovirus vaccine, which has been recognized as a high priority by the WHO since 2016 [33]. Among many vaccine candidates currently in development, at least four, across various formulations, have advanced to Phase 2 clinical trials or beyond (Table 1) [34]. Notably, two of these candidates have been evaluated or are currently being evaluated in pediatric populations.

**Table 1:** The four norovirus vaccine candidates under clinical evaluations

Company	Vaccine candidate	Adjuvant	Administration route	Antigen format	Antigen genotype	Status of trial
HilleVax	Hil-214, bivalent	Chitosan/MPLA, aluminum salt	Intramuscular, intranasal	Noroviral VLP	GI.1/GII.4	Phase 2b clinical trial
Zhifei	Longkoma, quadrivalent	Aluminum hydroxide	Intramuscular	Noroviral VLP	GI.1/GII.3/GII.4/GII.17	Phase 3 clinical trial
Vaxart	VXA-GI.1-NN, VXA-GII.4-NS, Mono- and bivalent	Adenovirus-expressed double-stranded RNAs	Oral	Adenovirus expressing noroviral VP1	GI.1/GII.4	Phase 2b clinical trial
Moderna	mRNA-1403, trivalent	NA	Intramuscular	Noroviral VP1-encoding mRNAs	GI.3/GII.3/GII.4	Phase 3 clinical trial

### The HilleVax Hil-214 Norovirus Vaccine

Hil-214, developed by HilleVax, Inc. (Table 1), is a virus-like particle (VLP)-based norovirus vaccine originally created by Takeda Pharmaceuticals International AG, where it was known as TAK-214 [35]. This bivalent vaccine contains VLPs derived from the prototype GI.1 Norwalk virus [36] and an artificially designed consensus VP1 sequence based on three predominant GII.4 variants isolated between 2002 and 2006 [37].

Phase 1 clinical trials demonstrated that the vaccine had a favorable safety profile, was well tolerated, and induced persistent serum antibody response [38–42]. Its protective effect in adults was further supported by Phase 2a studies. In one study, two intranasal doses of Hil-214 significantly reduced the frequency of acute gastroenteritis and viral infection following experimental challenge with the homologous GI.1 Norwalk virus [43]. In another study, two intramuscular doses of the vaccine reduced the incidence of vomiting and/or gastroenteritis after challenge with the GII.4 Farmington Hills/2002 variant [44]. These findings support that the Hil-214 vaccine provides protection against moderate to severe acute gastroenteritis caused by the homotypic noroviruses included in the formulation.

Noteworthy, the first Phase 2b field efficacy trial, which enrolled 4712 adults [45], demonstrated that the Hil-214 vaccine conferred 61.8% protection against moderate to severe norovirus-associated acute gastroenteritis, primarily caused by the heterotypic GII.2 genotype. This suggested cross-genotype protective efficacy. However, due to the limited number of homotypic norovirus gastroenteritis cases, the trial’s primary endpoint could not be fully evaluable.

Based on its success in adults, HilleVax expanded its focus to infants, the most vulnerable population. The company demonstrated that two and three intramuscular doses of HIL-214 were well-tolerated and elicited strong immune responses in infants aged four to six months [46]. A recent Phase 2b trial (ClinicalTrials.gov identifier: NCT05836012) [47] enrolled 2,824 infants, approximately 5 months old at the time of initial vaccination, to evaluate the vaccine’s effectiveness against moderate to severe acute gastroenteritis caused by homotypic GI.1 or GII.4 noroviruses.

Unfortunately, after two intramuscular doses, the vaccine showed only 5% efficacy and did not meet the primary endpoint. No clinical benefit was observed across secondary endpoints. Investigators believe that the appearance of multiple GII.4 variants during the trial may have contributed to the disappointing outcomes [47]. As a result, HilleVax has decided to discontinue further advancement of the vaccine for this age group. These findings have raised broader concerns about the challenges in developing an effective norovirus vaccine, particularly for young children, who are especially vulnerable to norovirus infection.

### The Zhifei Quadrivalent Norovirus Vaccine

Another norovirus vaccine currently under clinical evaluation in children and adolescents is a VLP-based quadrivalent vaccine containing four VLPs representing the GI.1, GII.3, GII.4, or GII.17 genotypes (Table 1). This vaccine, known as the Longkoma quadrivalent recombinant norovirus vaccine, is developed by Anhui Zhifei Longcom Biologic Pharmacy Co., Ltd. using a Pichia pastoris expression system. In earlier Phase 1 and Phase 2a trials (ClinicalTrials.gov identifier: NCT04563533), the vaccine’s safety, tolerability, optimal dose, and immunogenicity were evaluated in 580 participants ranging in age from six weeks to over 60 years, including infants, toddlers, adolescents, adults, and the elderly. The results from these trials provided a solid foundation for the ongoing Phase 3 efficacy study.

The current Phase 3 clinical trial (ClinicalTrials.gov identifier: NCT06524947) includes 6,600 children and adolescents aged from six weeks to 13 years, divided into three age groups: 1) 3,000 infants (6 to 23 months), 2) 2,200 toddlers (2 to 5 years), and 3) 1,400 children and adolescents (6 to13 years). Participants in each age group were randomly assigned in a 1:1 ratio to either the test group or the control group. The test group received three intramuscular doses of the vaccine at 30-day intervals to assess its protective efficacy, immunogenicity, and safety. The study began on July 27, 2024, and is expected to conclude by March 28, 2027.

Following the failure of the Hil-214 vaccine in infants [47], the results of this Phase 3 trial are particularly significant. They may either confirm the limitation of the Hil-214 vaccine or demonstrate that three doses of this quadrivalent formulation offers superior protection. Notably, the inclusion of GII.17 and GII.3 VLPs, genotypes associated with the current significant global disease burden (GII.17) [13–15] and common sporadic gastroenteritis in infants and young children (GII.3) [48], may enhance the vaccine’s effectiveness by increasing the number of norovirus-associated disease cases available for statistical analysis.

### The Vaxart Oral Norovirus Vaccines

Vaxart, Inc., a clinical-stage biotechnology company, has developed several oral norovirus vaccines in tablet form. These vaccines use recombinant, non-replicating adenovirus-based vectors that express the VP1 genes from noroviruses, along with a double-stranded RNA adjuvant [49, 50]. The formulation is designed to induce robust mucosal immune response in the intestine, which is critical for protecting against norovirus infection and disease.

Following a Phase 1 trial that demonstrated the safety, immunogenicity, and tolerability of the monovalent VXA-GI.1-NN vaccine in adults [49], a Phase 2b human challenge study [51] was conducted to evaluate its protective efficacy after a single oral dose. Key findings from the study [51, 52] included: 1) 29% protection against norovirus infection, 2) a statistically non-significant 21% reduction in symptomatic gastroenteritis, 3) an 85% reduction in viral shedding in stool and emesis, and 4) a robust increase in norovirus VP1-specific serum and mucosal antibodies, as well as antibody-secreting cells.

Given that both the VXA-GI.1-NN vaccine and the challenge virus contain the same GI.1 Norwalk virus VP1 gene, the observed vaccine efficacy, 29% against infection and 21% against gastroenteritis, was low. However, the 85% reduction in viral shedding is a noteworthy outcome, suggesting that the vaccine may play a meaningful role in limiting norovirus transmission.

Latest clinical trial data from Vaxart [53] indicated that its second-generation vaccine technology elicits significantly stronger antibody responses compared to its first-generation platform. Among other enhancements, the new bivalent vaccine, targeting both GI.1 (Norwalk virus) and GII.4 (2012 Sydney variant) genotypes, uses the same adenoviral vectors but expresses the VP1 genes from both strains to broaden protection across norovirus genogroups and genotypes.

Results from a recent Phase 1 clinical trial (ClinicalTrials.gov identifier: NCT05626803) [53] showed that the new bivalent vaccine induced a significantly higher levels of norovirus-blocking antibodies: 141% increase for GI.1 and 94% increase for GII.4, compared to those induced by the two first-generation constructs (VXA-G1.1-NN and VXA-G2.4-NS), respectively. These findings suggest the potential for improved protection against norovirus infection and disease.

### The Moderna mRNA Norovirus Vaccine

Moderna Inc., has developed a trivalent mRNA-based norovirus vaccine, named mRNA-1403, which encodes VP1 proteins from three norovirus genotypes: GI.3, GII.3, and GII.4. A combined Phase 1 and 2 clinical trial in younger adults (ages 18–49) and older adult (ages 60–80) was conducted (ClinicalTrials.gov identifier: NCT05992935), demonstrating good safety and tolerability. A single intramuscular dose elicited strong serum HBGA-blocking antibodies and norovirus VLP-binding antibodies specific to all three targeted genotypes in the two age groups.

Building on this progress, Moderna has initiated a multicenter, pivotal Phase 3 clinical trial, referred to as Nova 301 (ClinicalTrials.gov identifier: NCT06592794) [54], to assess the efficacy, safety, and immunogenicity of the mRNA-1403 vaccine. The trial aims to enroll approximately 25,000 participants globally, including 5,000 aged 18—59 and 20,000 aged 60 and older, the latter representing the population at greatest risk for severe illness outcomes such as hospitalization and death. This trial is expected to be completed by May 2027. The results of this study will provide critical insights into the efficacy of an mRNA-based approach to norovirus vaccination, particularly in protecting older adults, a key vulnerable population to norovirus infection.

## Major Challenges in Norovirus Vaccine Development

Despite the urgent need and recent advancements in both preclinical and clinical studies that have offered new insights into future success, developing a broadly protective norovirus vaccine remains a complex and multifaceted challenge. Several scientific, technical, and logistical obstacles continue to hinder progress in norovirus vaccine development.

### Genetic and Antigenic Diversity

A major challenge in norovirus vaccine development is the virus’s extensive genetic and antigenic diversity. As single-stranded RNA viruses, noroviruses evolve rapidly and are currently classified into 10 genogroups (GI–GX), with GI, GII, GIV, GVIII, and IX comprising viruses that infect humans. Within these five genogroups, more than 30 genotypes have been identified. Over the past few decades, the GII.4 strain has been the most prevalent globally and is known for frequent antigenic drift, similar to influenza viruses [55]. However, the recent emergence of the GII.17 strain, initially predominant in China [56] and some other parts of Asia [57], has since been detected as a dominant strain in Europe and North America [13, 15]. Whether GII.17 will eventually replace GII.4 as the predominant strain over the long term remains to be seen.

Given that multiple genotypes often co-circulate during a single epidemic season and that cross-genotype protection remains uncertain, norovirus vaccines are typically designed as multivalent formulations. These aim to provide broad and lasting protection by targeting the most commonly circulating genotypes and their variants. Special attention may also be given to strains that disproportionally affect high-risk populations. For example, GII.3, which frequently cause sporadic gastroenteritis in children [48], is included in both the Zhifei and Moderna vaccine candidates. Additionally, vaccines may need to be periodically updated to reflect shifts in the predominant circulating strains, a strategy similar to that used for influenzas vaccines.

### Lack of a Robust Cell Culture System and a Suitable Animal Model

Noroviruses cannot be easily grown in conventional cell culture systems. Although human intestinal enteroids have enabled limited norovirus replication, this system remains complex, expensive, and not yet scalable for widespread use. As a result, traditional vaccine approaches, such as live attenuated or inactivated vaccines that rely on efficient viral propagation, are not applicable. Current norovirus vaccine candidates instead rely on recombinant noroviral VLPs, viral vectors expressing the noroviral VP1 gene, and mRNA-based platforms encoding norovirus VP1 protein. Furthermore, the absence of a reliable and high-throughput culture system continues to hinder efforts to study viral biology, screen vaccine candidates, and assess neutralizing antibody responses.

Currently, no suitable small animal model exists that accurately mimics human norovirus infection and pathogenesis. Although gnotobiotic pigs and calves have been used for this purpose, they do not fully replicate the human infection process and are both costly and logistically challenging. Murine norovirus models are available but differ substantially from human noroviruses in terms of disease, pathogenesis and immune response. This limitation poses a significant barrier to preclinical development in the evaluation of vaccine protective efficacy.

### Poorly Defined Immune Correlates

A key unmet knowledge gap in norovirus vaccine development is the lack of well-defined immune correlates that reliably predict vaccine efficacy. Although serum noroviral VLP-binding antibodies and HBGA-blocking antibodies are commonly measured, their predictive value for clinical protection remains uncertain and may be influenced by prior exposure. For instance, the vaccine candidate Hil-214 (HilleVax) and VXA-GI.1-NN (Vaxart) have been shown to elicit high level of these antibodies, yet their protective efficacy against norovirus infection and disease has been reported as low [47, 51, 52].

Machine learning analysis of data from a recent Phase 2b clinical trial indicated that mucosal IgA response stimulated by the oral vaccine may serve as a robust correlate of protection [51]. However, our understanding of T-cell and innate immune responses, and how they interact with pre-existing immunity, remains limited. The absence of fully validated immune biomarkers continues to hinder the comparison of vaccine candidates and the accurate prediction of their efficacy.

### Other Challenges

Available data suggest that natural immunity following norovirus infection is generally short-lived and largely strain-specific, typically lasting less than two years. This raises important concerns about the durability of vaccine-induced protection. Clinical trial results have shown that antibody levels can decline within months post-vaccination, indicating that maintaining immunity may require booster doses and/or updated vaccine formulations.

The success of clinical trials evaluating vaccine efficacy in adults, but not in infants, for the Hil-214 vaccine candidate raise concerns about the potential difference in vaccine responses across age groups. Infants, who are likely immunologically naïve to norovirus, consistent with their high susceptibility to norovirus infection, may rely solely on vaccination for protection. This could explain the lower or absence protective efficacy observed in this age group. In contrast, adults have typically experienced multiple prior norovirus infections, and vaccination may act as a booster to pre-existing immunity. This disparity highlights a critical knowledge gap in understanding vaccine responses among infants, the elderly, and immunocompromised individuals, populations at highest risk for severe norovirus disease, compared to the healthy adults. Future studies are needed to address these issues and optimize vaccine strategies for vulnerable populations.

## Future Prospects and Path Forward

Current norovirus vaccine candidates, based on diverse platforms, such as recombinant VLPs, viral vectors, and mRNA, utilize different delivery routes, including intramuscular and oral administration, offering multiple strategies to combat norovirus. While intramuscular VLP-based vaccine have shown success against mucosal infection in the past (e.g., influenza and COVID-19), the failure of the Hil-214 vaccine (HilleVax) in infants [47] raises concerns about its efficacy in certain populations. The results of the ongoing Phase 3 trial of the Zhifei Longkoma vaccine in infants and similar age groups (ClinicalTrials.gov identifier: NCT06524947) will be important in addressing this issue.

Since norovirus is an enteric pathogen, Vaxart’s oral vaccines represent a more relevant vaccine approach to inducing mucosal immunity against intestinal norovirus infection. The monovalent VXA-GI.1-NN vaccine has shown encouraging results in a Phase 2b clinical evaluation against GI.1 Norwalk virus challenge [51, 52]. The efficacy of Vaxart’s second-generation bivalent vaccine (VXA-GII.4-NS/VXA-GI.1-NN) against other genotypes will be of great interest in future studies. Additionally, a live rotavirus-based bivalent oral vaccine is currently in preclinical development. This recombinant rotavirus, which expresses the norovirus VP1 protein, has been shown to induce both mucosal and systemic neutralizing antibodies in infant mice following oral administration [58]. This live bivalent vaccine candidate targeting the two most important enteric viral pathogens is worth further development.

Beyond traditional vaccine approaches, alternative prophylactic and therapeutic strategies may be needed for populations in which vaccines are less effective. For example, passive immunization could benefit immunocompromised individuals. In this context, avian immunoglobulin Y (IgY) targeting norovirus [59, 60] offers a low-cost option that could be formulated as a food supplement or oral capsule. The recent identification of epitopes that elicit broadly neutralizing antibodies against pandemic GII.4 variants or multiple GII genotypes [61] may guide the design of immunogens for producing broadly protective vaccines or IgY. Such IgY formulations could also provide protection for other vulnerable populations, including children, adolescents, the elderly, and travelers.

## Conclusion

Developing a broadly protective, safe, and durable norovirus vaccine remains a significant challenge, primarily due to the virus’s extensive genetic diversity, the lack of robust cell culture systems and suitable animal models, and the absence of well-defined immune correlates of protection. Nonetheless, recent advances in vaccine technologies, immunological methodologies, and clinical trial outcomes have generated renewed optimism. With sustained innovation, collaborative efforts, and strategic investment, the development of an effective norovirus vaccine appears increasingly within reach.

## Key References

- Ettayebi K, Kaur G, Patil K, Dave J, Ayyar BV, Tenge VR, Neill FH, Zeng XL, Speer AL, Di Rienzi SC, Britton RA, Blutt SE, Crawford SE, Ramani S, Atmar RL, Estes MK. Insights into human norovirus cultivation in human intestinal enteroids. mSphere. 2024;9(11):e0044824. Epub 2024/10/15. https://doi.org/10.1128/msphere.00448-24.
  - A useful enhancement of the current human norovirus culture system.
- Nyblade C, Yuan L. Virus Shedding and Diarrhea: A Review of Human Norovirus Genogroup II Infection in Gnotobiotic Pigs. Viruses. 2024;16(9). Epub 2024/09/28 22:44. https://doi.org/10.3390/v16091432.
  - A useful review discussing the only established animal model for human norovirus and disease.
- Cohen J. Why the ‘Ferrari of viruses’ is surging through the Northern Hemisphere. Science. 2025;387(6731):235-6. https://doi.org/10.1126/science.adv9834.
  - An insightful summary of the recent rise in GII.17 norovirus prevalence.
- Barclay L, Vinje J. Increasing Predominance of Norovirus GII.17 over GII.4, United States, 2022-2025. Emerg Infect Dis. 2025;31(7):1471-3. Epub 2025/06/02. https://doi.org/10.3201/eid3107.250524
  - An insightful observation highlighting the recent surge of the GII.17 genotype, surpassing the historically dominant GII.4 in prevalence.
- Mattison CP, Calderwood LE, Marsh ZA, Wikswo ME, Balachandran N, Kambhampati AK, Gleason ME, Lawinger H, Mirza SA. Childcare and School Acute Gastroenteritis Outbreaks: 2009-2020. Pediatrics. 2022;150(5). Epub 2022/10/25. https://doi.org/10.1542/peds.2021-056002.
  - A study providing additional evidence of the vulnerability of children and adolescents to norovirus infection.
- Saez-Llorens X, deAntonio R, Lopez-Medina E, Lopez P, Masuda T, Mendelman PM, Sherwood J, Baehner F, Borkowski A. Safety and immunogenicity of a bivalent norovirus vaccine candidate in infants from 6 weeks to 5 months of age: A phase 2, randomized, double-blind trial. Human vaccines & immunotherapeutics. 2025;21(1):2450878. Epub 2025/01/13. https://doi.org/10.1080/21645515.2025.2450878.
  - A study providing evidence supporting the safety and immunogenicity of norovirus vaccines in infants.
- HilleVax Reports Topline Data from NEST-IN1 Phase 2b Clinical Study of HIL-214 in Infants [Internet]2024 [cited July 6, 2025]. Available from: https://ir.hillevax.com/news-releases/news-release-details/hillevax-reports-topline-data-nest-in1-phase-2b-clinical-study.
  - This press release reports the failure of the most advanced norovirus vaccine candidate in its latest phase 2b clinical trial in infants.
- Flitter BA, Gillard J, Greco SN, Apkarian MD, D'Amato NP, Nguyen LQ, Neuhaus ED, Hailey DCM, Pasetti MF, Shriver M, Quigley C, Frenck RW, Jr., Lindesmith LC, Baric RS, Wei LJ, Tucker SN, Cummings JF. An oral norovirus vaccine generates mucosal immunity and reduces viral shedding in a phase 2 placebo-controlled challenge study. Sci Transl Med. 2025;17(798):eadh9906. Epub 2025/05/14. https://doi.org/10.1126/scitranslmed.adh9906.
  - A recent study demonstrating that an oral norovirus vaccine reduces viral shedding in a phase 2 clinical study.

## Data Availability

No datasets were generated or analysed during the preparation of this review article.
